# Translational Significance of Selective Estrogen Receptor Modulators in Psychiatric Disorders

**DOI:** 10.1155/2018/9516592

**Published:** 2018-10-08

**Authors:** Mohammad M. Khan

**Affiliations:** Department of Biochemistry and Molecular Biology, Faculty of Medicine, University of Zawia, P.O. Box 16418, Az-Zawiyah, Libya

## Abstract

Accumulating data from various clinical trial studies suggests that adjuvant therapy with ovarian hormones (estrogens) could be effective in reducing cognitive deficit and psychopathological symptoms in women with psychiatric disorders. However, estrogen therapy poses serious limitations and health issues including feminization in men and increased risks of thromboembolism, hot flashes, breast hyperplasia, and endometrium hyperplasia when used for longer duration in older women (aged ≥ 60 years) or in women who have genetic predispositions. On the other hand, selective estrogen receptor modulators (SERMs), which may (or may not) carry some risks of hot flashes, thromboembolism, breast hyperplasia, and endometrial hyperplasia, are generally devoid of feminization effect. In clinical trial studies, adjuvant therapy with tamoxifen, a *triphenylethylene* class of SERM, has been found to reduce the frequency of manic episodes in patients with bipolar disorder, whereas addition of raloxifene, a *benzothiophene* class of SERM, to regular doses of antipsychotic drugs has been found to reduce cognitive deficit and psychological symptoms in men and women with schizophrenia, including women with treatment refractory psychosis. These outcomes together with potent neurocognitive, neuroprotective, and cardiometabolic properties suggest that SERMs could be the potential targets for designing effective and safer therapies for psychiatric disorders.

## 1. Introduction

Over the past twenty years, the role of estrogens in neuroprotection and cognition has been extensively investigated in both rodents and human subjects [1–6]. The results of these investigations suggest that estrogen treatment induces robust neuroprotection and improves memory and cognition in model animals of various neurological disorders [1–3]. Likewise, in postmenopausal women, estrogen therapy may improve one or more domains of cognition including verbal memory, speech, abstract reasoning, and information processing [3–9]. Based on these findings, psychiatrists have used estrogens to treat cognitive abnormalities in patients with schizophrenia and other mental illnesses [10–18]. The outcomes of these clinical trial studies suggest that estrogen therapy may improve certain aspects of cognition and also reduces psychiatric symptoms in postmenopausal women with schizophrenia and bipolar mania [12–14, 17, 18]. However, long-term estrogen therapy carries significant risks of thromboembolism, hot flashes, breast hyperplasia, and endometrial hyperplasia in older postmenopausal women or those having genetic predispositions [19–21]. Additionally, its use in men is restricted because of the feminization effect, as well as in young or adolescent girls because of hypersensitization issue [19, 20]. Therefore, in recent years, efforts have been shifted toward selective estrogen receptor modulators (SERMs), which have shown potent estrogenic properties in the central nervous system (CNS). Although, SERMs may carry low risks of stimulating uterine or breast tissues but are devoid of feminization and hypersensitization effects [22–25].

SERMs are nonsteroidal estrogenic compounds derived from either the *triphenylethylene* or *benzothiophene* classes of compounds [23]. The members of the *triphenylethylene* class of SERMs include tamoxifen, clomiphene, toremifene, and GW5407, which are used primarily for the treatment/prevention of breast cancer. The members of the *benzothiophene* class of SERMs include raloxifene, arzoxifene, bazedoxifene, and lasofoxifene, which are used primarily for the treatment of osteoporosis; however, raloxifene has also been approved for the treatment of breast cancer in high-risk postmenopausal women [23–25]. Intriguingly, an overwhelming body of evidence suggests that both classes of SERMs also have potent neurocognitive and neuroprotective properties. Studies performed in normal and model animals suggest that SERMs may improve memory and cognition and may increase neurogenesis and synaptic plasticity in the injured brain [26–32]. In addition, they reduce oxidative stress and neuroinflammation [33–40], which are considered potent etiological factors in many CNS disorders.

In recent years, several clinical trials have been conducted using SERMs as adjuvant drugs to investigate their effects on cognition and psychopathological symptoms in psychiatric disorders including bipolar disorder [41–43], schizophrenia [44–46], and Alzheimer's disease [47]. Two of the SERMs, namely, tamoxifen and raloxifene, have shown promising results in bipolar disorder and schizophrenia, respectively. However, raloxifene intervention in Alzheimer's disease was not effective [47]. In this review, translational significance of the outcome of tamoxifen and raloxifene augmentation trials in psychiatric disorders and the possible mechanisms of actions underneath their neurocognitive, neuroprotective, and cardiometabolic properties are discussed.

## 2. Tamoxifen Augmentation Trials in Bipolar Disorder

Bipolar disorder is a mental illness that brings severe high and low moods and changes in sleep, energy, thinking, and behavior. A manic episode occurs when patients have periods in which they feel overly excited and confident. They may show sometimes irritability and impulsive or reckless behavior and may experience delusions or hallucinations [48–50]. In addition, bipolar patients also display impairments in various cognitive domains including verbal memory, working memory, psychomotor speed, verbal fluency, attention, speed of information processing, executive function/reasoning, and problem solving [48, 51, 52]. These cognitive impairments can be identified in all phases of the disorder but are more frequent during manic episodes.

Treatment of bipolar disorder is challenging because the drugs that are used may be effective for a specific phase but may not be effective for other phases or they may even worsen the outcome of the illness [48, 53–57]. However, in the last 10–15 years, several synthetic agents have been developed that are used for the treatment of different phases of bipolar disorder including acute mania, acute depression, and relapse prevention [53, 56, 57]. The advantage of using tamoxifen in augmentation therapy is that it can be effective in all phases of the disease, although evidence suggests that it has a more profound effect on manic episodes [41–43].

A preliminary study conducted by Bebchuk et al. showed that addition of 60–80 mg/d tamoxifen to regular doses of antidepressants significantly reduced the frequency of manic episodes in men and women with bipolar disorder [58]. Several subsequent studies also observed a significant reduction in acute mania by tamoxifen used at a dose of 40 mg/d [58–61]. However, one study could not reproduce their earlier findings in a large four-week randomized controlled trial performed using the same dose of tamoxifen [62]. The lack of tamoxifen effectiveness in their study might be due to its low dose and large sample size used. Contrary to this, several other randomized, double-blinded clinical trials conducted in women and children have reported significant reduction in the symptoms of mania by adjunct tamoxifen used at different doses starting from 40 mg/d up to 120 mg/d and the treatment was well tolerated for up to four weeks without inducing any serious adverse effect [63–65].

A meta-analysis while reviewing electronic data on all the randomized controlled trials also found that, in most cases, tamoxifen adjuvant therapy effectively reduced the frequency of manic episodes in bipolar patients [66]. This study suggests that tamoxifen can be considered an effective adjuvant in the treatment of manic bipolar patients. However, most of these clinical studies were piloted for short durations ranging from one to four weeks; therefore, conclusion regarding the efficacy and safety of tamoxifen use for longer periods and its effect on cognition warrants further studies with larger sample sizes and longer follow-up duration.

## 3. Raloxifene Augmentation Trials in Schizophrenia

Schizophrenia is a neurodevelopmental disorder that displays variable degree of cognitive deficit along with positive and negative symptoms of psychosis. The most common positive symptoms include hallucinations, delusions, and thought disorder, whereas most prevalent negative symptoms include apathy, blunted effect, and social withdrawal [67–69]. Cognitive deficit may be associated with a deficit in attention, working memory, verbal speech, executive function, and social cognition. In fact, cognitive deficit has been recognized as the potential risk factor in schizophrenia because it is generally present long before the onset of psychosis and becomes more severe as the illness advances [70–73]. Cognitive deficit adversely affects the ability of individuals to achieve academic standard, employment, and social status; therefore, improving cognition is considered a crucial therapeutic domain in integrating people with schizophrenia into the working environment and social life [74, 75].

Although over the last five-six decades, more than fifty antipsychotic drugs have been developed and used in various combinations, they have failed to improve cognition in schizophrenia. Also, their effectiveness to reduce psychopathological symptoms, especially negative symptoms, is far from convincing; instead, the risk of cardiometabolic morbidity and mortality is greatly increased after treatment is initiated [72, 75–82]. Additionally, some clinical trials performed using different supplementation strategies have not been very effective as they reduced mostly positive symptoms but not the negative symptoms and cognitive deficit [83–85]. Consequently, treating cognition and negative symptoms in schizophrenia is still an unresolved issue and the calls are growing for more effective therapies [74, 75, 78, 79, 83–85]. However, some clinical trial studies in which ovarian hormones (estrogens) were used as an adjuvant to antipsychotic drugs have shown promising results in schizophrenia. In one study, adjuvant estradiol treatment improved scores on comprehension of metaphoric speech without affecting verbal ability and word fluency [86]. Comprehension of metaphoric speech, a main feature of thought and language, is impaired in schizophrenia. In another study, adjuvant estradiol treatment effectively reduced PANSS positive, general, and total symptoms but did not reduce negative symptoms and cognitive deficit in women with treatment-resistant schizophrenia [13]. However, there is evidence suggesting that adjuvant estrogen treatment may improve certain aspects of cognition including memory, verbal fluency, and visual attention/psychomotor speed in chronic schizophrenia women of childbearing age [87]. These results are also supported by the clinical findings in which low circulating estrogen levels were associated with more sever negative symptoms and reduced cognitive performance, especially, verbal performance and executive functioning in women suffering from chronic schizophrenia [88]. Moreover, there is evidence suggesting that apart from positive, general, and total symptoms, adjuvant estrogen treatment may also reduce negative symptoms in women with schizophrenia [89–91]. Taken together; the above findings suggest that adjuvant estrogen therapy could be effective in reducing cognitive and psychopathological symptoms in schizophrenia. However, as discussed above, estrogen treatment may have some serious limitations and risks involved, which limits its use on a wider scale [19–21]. Consequently, in recent years, several clinical trials were conducted using raloxifene as adjuvant drug in treating cognition in schizophrenia, as discussed below. The results of these trial studies suggest that use of raloxifene is not only safer but may also lower the dose of antipsychotic drugs to achieve the same therapeutic outcome and may reduce side effects associated with long-term antipsychotic treatment.

Initial clinical trial studies with raloxifene adjuvant therapy were mainly performed in postmenopausal women with schizophrenia [44–46, 92–94]. In one study, two groups of patients treated with 60 and 120 mg/d doses of raloxifene as an adjuvant to antipsychotic drugs for 12 weeks showed significant reduction in PANSS total score and the general symptom score. The patients treated with higher raloxifene dose showed greater improvement [44]. Another placebo-controlled study in which postmenopausal women randomized to 60 mg/d raloxifene adjunct therapy showed significant reduction in the positive, negative, and general psychopathological symptoms after 12 weeks of treatment compared with women receiving placebo [45]. The same group recently confirmed their findings in a larger sample and longer duration (24 weeks) of treatment [46], which suggests that raloxifene augmentation is an effective strategy for treating positive, negative, and general psychological symptoms in postmenopausal women with schizophrenia. Other clinical trial studies have also reported similar reduction in symptoms after raloxifene adjuvant therapy [92, 93]. However, in one study, Iranian postmenopausal women with schizophrenia, when treated with 120 mg/d raloxifene as an adjunct to risperidone (6 mg/d), showed improvement in positive symptoms only, whereas negative and general psychopathology symptoms did not improve [94].

Recently, raloxifene adjunct therapy has also been successfully tested in young men and women with schizophrenia including women with treatment-resistant psychosis [95, 96]. In a randomized, double blind, placebo-controlled study, forty-six male schizophrenia patients were treated with either 120 mg/d raloxifene or placebo in addition to risperidone (6 mg/d) for eight weeks. The patients showed significant improvement in negative symptoms, general symptoms, and PANSS total score but not the positive symptoms [95]. Also in young women with treatment-resistant schizophrenia, treatment with 120 mg/d raloxifene as an adjuvant significantly reduced PANSS total and general symptom scores [96]. In other studies also, treatment with the same dose of raloxifene reduced PANSS general and positive symptom scores on hallucinatory behavior, agitation, and restlessness and also improved certain domains of cognition including attention, disorganized behavior, and sociooccupational functioning, and in some cases, therapeutic outcome was steady and maintained even after the dose of raloxifene was reduced to half [97, 98]. These results suggest that raloxifene adjuvant therapy can improve certain aspects of social and nonsocial cognition. While a recent study did not observe any effect of raloxifene on mood and cognition in young schizophrenia women [96], other studies have indeed reported a positive effect of raloxifene adjuvant therapy on one or more domains of cognition.

In a 12-week randomized, placebo-controlled study, addition of 60 mg/d raloxifene to a regular antipsychotic dose improved verbal learning with no significant effect on long-term memory or recognition [99]. The authors also replicated this effect in a clinical case study in which a postmenopausal woman, treated with 60 mg/d raloxifene adjuvant, showed improvement in psychopathology and executive functions of cognition [100]. In another thirteen-week trial, addition of 120 mg/d raloxifene to their routine antipsychotic medications significantly improved attention/processing speed and memory in both men and women with schizophrenia [98]. Functional magnetic resonance imaging studies performed in male and female schizophrenia patients have shown that raloxifene treatment can also improve probabilistic association learning and emotional face recognition (a form of cognitive process that is impaired in schizophrenia) with concomitant increase in neuronal activity in the associated brain regions [101].

Recent meta-analyses performed on the outcome of all the raloxifene trial studies in schizophrenia concluded that raloxifene as an adjuvant is effective against all domains of schizophrenia psychosis, i.e., positive, negative, and general symptoms; however, the extent to which the symptom scores are reduced in each domain varies. Raloxifene may also improve cognition in both male and female schizophrenia patients including postmenopausal women [102–104]. This may be a very promising outcome because various imaging and histological studies suggest that negative symptoms and cognitive impairment are more strongly associated with the structural abnormalities in the brain than with positive symptoms [105–107]. While further clinical trials are needed to replicate the effect of raloxifene on cognition, initial evidence that raloxifene improves cognition and reduces negative symptoms even in treatment-resistant psychosis may suggest an improved structural plasticity and functional connectivity of the brain, which are otherwise reduced in schizophrenia patients [108–110].

## 4. Mechanism of SERM Actions in CNS

Data from various *in vitro* and *in vivo* animal studies suggests that SERMs have a peculiar mode of action. They can act as ER agonists in CNS tissues and as ER antagonists in non-CNS tissues. Acting as ER agonists, SERMs induce a number of estrogenic effects in the brain, which may regulate neuroprotection, memory and cognition, and the underlying brain connectivity [22, 26–32]. It is intriguing how various SERMs produce ER agonist or antagonist effects and how these effects relate to the clinical outcomes in various brain disorders. Whether a SERM is effective as an ER agonist or antagonist in a particular tissue depends upon several factors including the ER subtypes present, conformation of the ERs induced by the SERM, level, types, availability of coactivators and corepressors in the tissue, and types of coactivators and/or corepressors recruited to form the ER complex and the degree of interactions [22, 29, 111–114].

Although SERMs were initially thought to interact with classical ERs, that is, ER*α* or ER*β*, recent reports suggest that they also interact with transmembrane G protein-coupled estrogen receptor-1 (GPER-1) in CNS [29, 111–114]. Thus, as of now, SERMs can interact with all the three subtypes of ERs in neurons and glia and can initiate both genomic and nongenomic signaling including activation of the cAMP/PKA, MAPK/ERKs, PI3K/Akt, JAK/STAT3, Wnt/*β*-catenin/GSK3*β*, and Nf-KB pathways [28, 29, 114–116]. All these signaling pathways regulate memory and cognition, neuroprotection, and brain regeneration process. However, the extent to which these signaling pathways are activated by different SERMs may differ, because of their differential affinity for ERs. It has been observed that the affinity of raloxifene is 4-fold higher (relative to estrogen) for ER*α* than ER*β*, whereas the affinity of tamoxifen is similar for both receptors [117,118].

**Figure 1:**
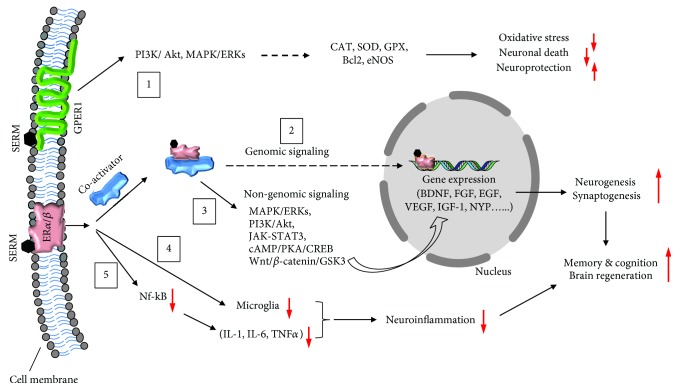
Possible signaling mechanisms of SERM actions in neurocognition and neuroprotection. Over the last few years, others and we have shown that SERMs can mediate their actions by initiating genomic (gene expression) and/or nongenomic signaling that involve kinases and phosphatases. In CNS tissues, SERMs can bind and activate both classical estrogen receptors-*α* and *β* (ER*α* or ER*β*) as well as nonclassical transmembrane G protein-coupled ER (GPER1). Via agonist action at GPER1, SERMs can activate the PI3K/Akt and MAPK/ERK pathways (Figure 1, box 1), which have been shown to be involved in neuroprotection and reduction of oxidative stress and neuronal cell death by increasing the expression of antioxidant enzymes (CAT, SOD, GPx, and eNOS), Bcl2, and other trophic factors. Via agonist action at the classical ER*α* or ER*β*, SERMs can activate gene expression/genomic signaling (Figure 1, box 2) of various growth factors and proteins involved in synaptic plasticity, neurogenesis, memory, and cognition. SERMs can also enhance interaction of ER*α* or ER*β* with MNAR/PELP1, a scaffold/coactivator protein highly expressed in neurons and astrocytes [165,166]. The resultant ER-MNAR/PELP1 complex can then initiate nongenomic signaling by activating the PI3K/Akt, MAPK/ERK, and Wnt/*β*-catenin/GSK3*β* signaling pathways (Figure 1, box 3). These pathways have been shown to regulate neurogenesis, synaptogenesis, and cognitive behaviors in the normal and model animals of diseases. SERMs can reduce brain inflammation by acting on astroglia via ER*α* or ER*β*. They can reduce microglia proliferation (Figure 1, box 4) as well as production of inflammatory cytokines and chemokines including IL-1, IL-6, and TNF*α* via inhibition of nuclear factor-kappa-B (Nf-KB) transactivation (Figure 1, box 5). Inhibition of this pathway has been shown to induce neuroprotection and reduce neuronal cell death in various cellular and animal models of brain injury. Red arrows indicate increase (upward) or decrease (downward) in the magnitude of response by SERMs.

After interacting with cell membrane ERs, SERMs can activate numerous cell signaling pathways as mentioned above and are shown in Figure 1. While that is the case in general, tamoxifen-induced inhibition of protein kinase C (PKC) is considered a prime reason for reduction in manic episodes in bipolar patients [41–43, 58, 60, 63]. However, the downstream effects of PKC inhibition may involve several other changes including alteration in synaptic plasticity/transmission, oxidative stress, neuroinflammation, and calcium and glutamate toxicity [28, 30, 33, 120–122]. Tamoxifen may reduce calcium toxicity directly by inhibiting calcium channels like raloxifene and estradiol [120–122]. However, whether calcium toxicity is the major cause of mania and whether its reduction by tamoxifen is a possible mechanism involved in its beneficial effects in bipolar patients remain to be investigated. Interestingly though, calcium channel-linked SNPs have been identified as risk alleles [123, 124] and certain types of calcium channel blockers have been found effective in reducing manic episodes in bipolar patients [125, 126]. Further, animal studies have shown that both tamoxifen and raloxifene can prevent various receptor-mediated disruptions in prepulse inhibition [127], which is a measure of sensorimotor gating that is reduced in bipolar disorder, schizophrenia, and other psychiatric diseases [128–130].

Apart from PKC signaling involvement in the action of tamoxifen, several other signaling pathways are also activated by both tamoxifen and raloxifene as depicted in Figure 1. Raloxifene, in particular, has been studied extensively in CNS tissues, where it has been shown to activate the cAMP/PKA, MAPK/ERKs, PI3K/Akt, JAK/STAT3, Wnt/*β*-catenin/GSK3*β*, and Nf-KB pathways under various *in vitro* and *in vivo* experimental conditions [28–30, 114–116]. By activating these signaling pathways, raloxifene has been shown to regulate structural and functional plasticity underlying memory and cognition, neuroprotection, neurogenesis, oxidative stress, and neuroinflammation in the normal and model animals of CNS disorders [28–40]. Although tamoxifen also regulates most of these parameters, some evidence suggests that raloxifene may be more effective, probably, because of its high affinity for ER*α* compared to ER*β*. However, the two ER subtypes may have different functional implications. In the neuroprotective and anti-inflammatory properties of raloxifene, ER*α* may play a major role as evidence suggests that ER*α* but not ER*β* is involved in the neuroprotective and anti-inflammatory properties of 17*β*-estradiol [119]. This has also been reflected in some *in vitro* and *in vivo* animal model studies, including our own, in which raloxifene has been found more effective than tamoxifen [30, 35]. On the other hand, evidence suggests that ER*β* may play a major role in synaptic plasticity, memory, and cognition compared to ER*α* [131–133]. In conclusion, the cognitive and psychopathological outcome of raloxifene adjuvant therapy in schizophrenia may be due to a combined effect of ER*α* and ER*β* activation. However, further studies are needed to investigate the relative contribution of the two ER subtypes and also GPER-1 in the therapeutic effectiveness of raloxifene.

## 5. SERMs Reduce Oxidative Stress and Neuroinflammatory Cues

Mounting evidence suggests that prolonged psychological and social stresses can increase the levels of reactive oxygen species (ROS), proinflammatory chemokines, and cytokines produced by activated microglia [134–140]. Elevated ROS has been linked to several brain pathologies including the loss of parvalbumin-containing interneurons (reduced neurogenesis) and oxidation of lipids, nucleic acids, and proteins [141–147]. Similarly, excess of proinflammatory chemokines and cytokines such as IL-1*β*, IL-6, and TNF*α* has been found to affect development, morphology, and the firing rate of neurons. It has been suggested that chronic inflammation of the brain can also lead to interneuron loss, NMDA receptor hypofunction, dopamine deregulation, and white matter abnormalities, consequently impairing cognitive and noncognitive behaviors including olfaction social interaction, reproduction, and energy balance. These behavioral abnormalities together with the above-stated neurochemical and neuroanatomical pathologies have been reported in bipolar and schizophrenia patients [141, 146–151].

Reduction in oxidative stress and reduction in neuroinflammation are the two additional potential mechanisms of SERMs, which may contribute to their effectiveness in psychiatric disorders. A number of laboratories have investigated the antioxidative and anti-inflammatory effects of SERMs, in both *in vitro* and *in vivo* experimental studies [28, 31–40, 152–157]. While both tamoxifen and raloxifene have been shown to reduce oxidative stress by increasing expression of various proteins and enzymes involved in antioxidant defense [152–157], tamoxifen under certain conditions may in fact increase oxidative stress, although, by different mechanisms [158]. The antioxidative effect of raloxifene includes regulation of B-cell lymphoma regulator protein (Bcl-2), catalase, superoxide dismutase, and glutathione peroxidase gene expression and the level of reduced glutathione in the brain [33–40, 152–157]. Additionally, raloxifene has been shown to increase mRNA expression of apurinic/apyrimidinic endonuclease/redox factor-1 suggesting that it may protect against ROS-induced DNA damage. Increased ROS-induced DNA damage has been reported in the brain tissue of schizophrenia patients [159].

The anti-inflammatory effect of SERMs may be mediated by multiple pathways including the reduction in the levels of IL-6, IL-1*β*, IP-10, and TNF*α* via suppression of microglia activation [35–39]. Additionally, raloxifene has been shown to block IL-1*β*-induced Nf-KB transactivation (phosphorylation of p65) and expression of the CCL20 (chemokine (C-C motif) ligand-20) protein in the reactive astrocytes in an animal model of autoimmune encephalomyelitis, a chronic inflammatory condition [40]. Thus, raloxifene can be considered an effective antioxidative and anti-inflammatory agent, perhaps more potent than estrogen and tamoxifen [35].

## 6. Safety of SERMs

Data from various long-term clinical trial studies in which postmenopausal women were treated for breast cancer and osteoporosis suggests that SERM therapy carries some risks such as hot flashes, leg cramps, and venous thromboembolic events. Tamoxifen treatment may also carry the additional risks of hyper-proliferation of the uterine and endometrial tissues, and may be cognitive decline in older women, especially, when it is used for longer duration [25, 160–162]. However, these risks are observed in older women and after years of treatment [25, 160, 161]. These adverse effects have not been reported in young women or in postmenopausal women who did not have previous history of complications [160, 161]. In conclusion, most of these analyses suggest that raloxifene has a favorable safety profile and its adverse effects, if any, can be reduced/minimized by changing dosing time and duration without affecting its therapeutic efficacy [162].

## 7. Future of SERMs in Psychiatric Disorders

Evidence for therapeutic effectiveness of SERMs in psychiatric disorders is emerging. SERMs can improve the clinical response of psychotropic drugs in patients with bipolar disorder and schizophrenia. While tamoxifen adjuvant therapy in bipolar patients requires additional studies on its safety for long-term use, raloxifene because of its favorable safety profile can be used safely in the long-term management of schizophrenia. Both of these SERMs have also been shown to prevent the development or delay the onset of cardiometabolic complications including diabetes, obesity, and atherosclerosis (reviewed in [163, 164]), which are serious adverse effects often present from the early phase of illness in both schizophrenia and bipolar patients and become more severe after treatment with psychotropic drugs [80–82]. Therefore, use of SERMs may improve therapeutic efficacy of psychotropic drugs and the quality of life of psychiatric patients after treatment. Unlike tamoxifen, raloxifene has been found to improve cognition or delay the onset of cognitive decline in postmenopausal women on osteoporosis therapy. Therefore, further studies on the potential of raloxifene to improve cognitive behaviors would be very crucial because, currently, there are no effective drugs available to improve cognition in schizophrenia.

Additional advantages of using raloxifene in schizophrenia would be a negligible or no risk of feminization in men and hypersensitization in adolescent girls or younger women that may be observed with estrogens. Because of this advantage and noteworthy brain- and behavior-repairing properties, raloxifene (or other more effective *alike* SERMs) provides an option for early intervention in schizophrenia, which might be more effective in correcting brain pathologies that lead to the development of cognitive deficit and psychosis in high-risk adolescents/individuals. Since evidence suggests that raloxifene adjuvant therapy may also reduce negative symptoms, which are more prominent in male compared to female schizophrenia patients, therefore, addition of raloxifene may enhance the potency of antipsychotic drugs to reduce negative symptoms more effectively in male schizophrenia patients.
